# Characteristics of the ideal hospitalist inpatient care program: perceptions of Canadian health system leaders

**DOI:** 10.1186/s12913-021-06700-0

**Published:** 2021-07-04

**Authors:** Vandad Yousefi, Elayne McIvor

**Affiliations:** 1 Fraser Health, Central City Tower, Suite 400, 13450—102nd Avenue, Surrey, British Columbia V3T 0H1 Canada; 2 Catalyst Consulting Inc, Vancouver, British Columbia Canada

**Keywords:** Hospital Medicine, Hospitalist, Program Implementation, Perceptions, Inpatient Care, Hospital Administration

## Abstract

**Background:**

Despite the growing prevalence of hospitalist programs in Canada, it is not clear what program features are deemed desirable by administrative and medical leaders who oversee them. We aimed to understand perceptions of a wide range of healthcare administrators and frontline providers about the implementation and necessary characteristics of a hospitalist service.

**Methods:**

We conducted semi-structured interviews with a range of administrators, medical leaders and frontline providers across three hospital sites operated by an integrated health system in British Columbia, Canada.

**Results:**

Most interviewees identified the hospitalist model as the ideal inpatient care service line, but identified a number of challenges. Interviewees identified the necessary features of an ideal hospitalist service to include considerations for program design, care and non-clinical processes, and alignment between workload and physician staffing. They also identified continuity of care as an important challenge, and underlined the importance of communication as an important enabler of implementation of a new hospitalist service.

**Conclusions:**

Most hospital administrators and frontline providers in our study believed the hospitalist model resulted in improvements in clinical processes and work environment.

## Background

Globally, multi-morbidity—defined as the presence of two or more chronic medical conditions—has been rising, with a prevalence in the general population that ranges between 9.7 and 90.5% across a range of high and low income countries [1]. In Canada, the prevalence of patients with multimorbidity is estimated to be between 10 and 25% and has been increasing. [2] Patients with multi-morbidity utilize higher levels of healthcare resources, including hospitalizations [3, 4]. Once hospitalised, multi-morbid patients have longer length of stay [5], higher mortality [6, 7] and 30 day readmission [8].

For many healthcare systems, particularly in the United States and Canada, meeting the care demands for an increasingly older and complex population coincides with concurrent constraints on available resources (such as acute care beds, qualified healthcare professionals, and funding to support increasingly complex technologies) [9]. In addition to finding ways to increase efficiency, many acute care organizations have also had to fundamentally change the way they organize the manner by which they deliver inpatient care services.

One strategy has been to create teams of professionals from various disciplines, lead by physicians with generalist training backgrounds and skill sets called “hospitalists” who dedicate their clinical practice to caring for patients in an acute care setting [10]. Hospitalists work as part of Hospital Medicine (HM) programs, which can incorporate non-physicians (such as Nurse Practitioners and Physician Assistants) and work closely with other health professionals. While the hospitalist model first emerged in North America, it has been rapidly adopted in many countries over the past two decades, [11–14]. A number of systematic reviews have suggested that hospitalist care is associated with reductions in length of stay (LOS) and cost of hospitalization along with improvements in patient satisfaction [15, 16]. Similarly, studies in the United States (US) have shown widespread acceptance of the hospitalist model among hospital administrators [17, 18].

### Hospital care in Canada

Historically, family physicians (FPs) in Canada provided medical oversight for the care of the majority of hospitalised patients as the Most Responsible Providers (MRP) in the acute carer setting. As MRPs, these FPs addressed the day to day medical needs of patients and coordinated with other providers (such as specialists, allied health professionals and nursing staff). [12]. These “full service” family physicians practiced a mix of both inpatient and outpatient care, and were compensated directly from provincial publicly funded health insurance plans through fee-for-service (FFS) payments [19]. However, despite the increasing complexity and multimorbidity of hospitalized patients and the growing need for better efficiency and care coordination in the acute care setting, FFS structures across Canada failed to keep up with the increasing workload associated with inpatient care. As a result, FFS payment models significantly under-valued inpatient care and physician effort needed to deliver it [20]. This contributed to a steady decline in the number of physicians willing to provide hospital care over time [21].

In order to attract MRPs, healthcare organizations across Canada created hospitalist programs, and to make them viable, provided various forms of additional compensation (commonly referred to as “top ups”). In many institutions, these hospital medicine programs quickly expanded to care for the bulk of general medicine hospitalized patients [22, 23]. In little over two decades, organizations that were accustomed to seeing their patients cared for by a range of physicians (such as community family physicians, internists and other specialists) at no direct cost, found themselves in a position where they increasingly needed to reallocate funds form their “global budgets” to compensate physicians for delivering inpatient care [10, 17].

### Acceptance of hospitalist programs

Hospitalist programs are now prevalent in Canada [12]. Despite this, it is unclear how Canadian medical and administrative health system leaders perceive their potential benefits. Anecdotal evidence suggests that some leaders may not appreciate the value that hospitalists can bring to their institutions [24]. This may be partly due to a strong belief in the merits of traditional models for inpatient physician coverage (such as full service family medicine [25]), or the financial burden of subsidizing hospitalist physician compensation and program administrative costs [17].

Physicians and administrators perceive their roles and the challenges of delivering healthcare using different mindsets and mental models [26]. Understanding how health system leaders assess the value of their hospital medicine programs can help hospitalist physicians appreciate the other side’s perspectives and allow them to identify common priorities, in turn enabling closer working relationships. At the same time, hospital leaders who are contemplating implementing new programs can benefit from the insights of their peers who have already gone through a similar process. This can allow for a proactive approach to the design of the new inpatient service and upfront negotiations with physicians to set clear expectations and mutual accountabilities.

To our knowledge, there are no published data on the perceptions of Canadian health system leaders on the value of the hospitalist model of care, the characteristics of an ideal hospital medicine inpatient care program, and potential strategies to optimize the hospital medicine service lines. We aimed to understand the opinions of a wide range of hospital administrators and frontline clinicians in an integrated, regional healthcare system in Western Canada about the ideal inpatient care model, the attributes and characteristics of an ideal hospitalist service, challenges associated with the new model and potential solutions.

## Methods

### Setting

Fraser Health (FH) Authority is the largest health delivery system in British Columbia, and is responsible for a range of acute and ambulatory services to 1.8 million residents [27]. Between 2016 and 2018, FH implemented new hospitalist services in 3 of its acute care facilities: a small community hospital in a semi-rural town, a medium-sized community hospital in a growing community, and a large referral centre. We therefore designed and conducted a program evaluation to understand the perspectives of a wide array of stakeholders about their newly implemented hospital medicine services.

### Interviews

As part of a comprehensive program evaluation of the three new hospitalist services, we conducted semi-structured interviews of a purposive sample of system leaders. The semi-structured interview approach was selected given interest in collecting data on a consistent set of questions across hospitalist sites, while also allowing for in-depth exploration of emergent areas through the use of probes and follow-up questions. The approach is also the most frequently used qualitative data source in health services research and was responsive to the researchers’ needs to explore interviewees’ perceptions, thoughts, feelings and beliefs about the topic at hand [28].

We invited a purposeful sample of senior and mid-level administrators and other frontline professionals who either worked locally at the impacted facilities or at the regional headquarters. A purposeful sampling strategy was adopted given interest in gathering data from: information rich cases; key administrators who were particularly knowledgeable about the design and implementation of their hospitalist programs; and a variety of frontline worker perspectives. We also employed snowball sampling by asking interviewees to identify other key contacts that could enhance the evaluation. Finally, we invited respondents to an online survey sent to staff at the three hospital sites to participate in a follow-up interview on a voluntary basis. The interviewee sample size was based on the point at which thematic saturation was reached. After completing 38 interviews, saturation was achieved since additional interviewees were not producing new thematic information.

The interviews were performed in-person or over the telephone and on average lasted 42 minutes. These were audio-recorded and subsequently transcribed for analysis. Informed consent was obtained from all interviewees at the outset, and all interviewees were informed that they could withdraw at any point in time. The transcripts were repeatedly read and initial thematic codes were generated. These were organized into main and sub-categories, and quantified. Themes were subsequently interpreted. We excluded one interviewee since they were not familiar with the hospitalist model at their site. Institutional Research Ethics Board of Fraser Health Department of Research and Evaluation exempted the need for ethical approval for the study. All methods were performed in accordance with the relevant guidelines and regulations.

## Results

### Interview participants

Table 1 summarizes the characteristics of the interview participants. Hospital administrators (including physician leaders) comprised the largest group of respondents, followed by frontline nurses, other professionals and practicing physicians.

**Table 1:** Interview participants by role (n = 38)

Role of interview participants	
Hospital administrators (including physician leaders; e.g. Program directors, Operations Managers, Heads of Departments, Executive Directors)	39% (n = 15)
Nursing staff	16% (n = 6)
Allied healthcare professionals	13% (n = 5)
Patient Care Coordinators	13% (n = 5)
Hospitalist and non-hospitalist generalist physicians (general practitioners or family physicians)	11% (n = 4)
Non-hospitalist specialist physicians (general internists, surgeons, emergency medicine specialists etc.)	8% (n = 3)

### The ideal inpatient care model

At all three sites, the introduction of the hospital medicine service was a great departure from the historical inpatient care model where community-based family physicians provided MRP care to hospitalised patients. Given the magnitude of this change, we asked interviewees to outline their ideal inpatient care model without considering resource limitations. Most interviewees (71%; n = 27) indicated that in their view, the hospitalist model was the ideal inpatient care system. For these respondents, the onsite availability of hospitalists was a key consideration that not only had a positive impact on quality of care, but also facilitated better communication with non-physician team members and higher quality inter-professional collaboration.

“Hospitalist on-site presence improves timeliness of decisions. This was a challenge in the past…discharges were delayed because GPs would already be back at their offices.”

For other interviewees (33%; n = 9), the ideal inpatient care model involved community-based family physicians who acted as MRPs for their own patients. These interviewees hypothesized that pre-established relationships between patients and their family physicians and the resulting continuity of care would be important from a patient perspective. At the same time, most of these respondents recognized that such a care model was no longer sustainable in many communities.

“The model of care we had 20 years ago had a lot of merit in terms of patient care. The problem is that over the years care has become more complex. It’s not possible to do efficient work when you are running an office and trying to run a hospital practice on the same day.”

Two interviewees reported that they were unsure about the ideal inpatient care model.

### Characteristics of an ideal hospitalist service

We asked respondents to provide further details about design and operational characteristics that an ideal hospitalist model would have. They described elements of hospitalist program structure (e.g. staffing considerations, work schedules, daily organization of workflow), clinical and non-clinical care processes (e.g. communication between hospitalists and others, access to consultants), and considerations with respect to workload demands and staffing levels. Table 2 summarizes the emerging themes and provides illustrative quotes. Note that only some of the interviewees described ideal hospital model characteristics (n = 27).

**Table 2:** Elements and characteristics of ideal hospitalist programs as reported by interviewees (n = 27)

Program domain	Ideal characteristics	Illustrative quote
Model design	Committed group of providers who are available 24/7 Rotating, unit-based models of care to improve efficiency Creation of teams of providers including a mix of hospitalists, specialists, nurse practitioners, nursing staff and allied health care Schedule designed to maximize continuity of care Flexibility and responsiveness to change	“Being on-site lends itself to better communication because they’re accessible. Hospitalists always answer the phone, but the GPs don’t always since they may be with other patients” “In a perfect world, they shouldn’t have to run around to all the departments. They should be unit-based, to improve efficiency. And they can rotate around so that nobody gets stuck with medical all the time; no body gets stuck with surgical all the time” “It should be team-based care, so teams of hospitalists, internists, NPs, nurses, and so on. Each can take patients depending on their complexity. Management would be needed to ensure the appropriateness of assignment” “Most are interested in the team and using each members’ strengths” “Less rotation between hospitalists so patients have a consistent doctor throughout their stay” “Changing doctors during the same admission for a patient. Continuity of care affected, more room for error” “We are so flexible. If we see something that’s not working, we change it as a group. We have a meeting every month to go over things and see what’s working, what’s not working, what the team wants”
Clinical and non-clinical processes	Easy access to consultants, particularly when managing complex patients Tools and supports for rapid communication and consultation with community-based FPs Easy identification of patient assignment to individuals (i.e. which hospitalist is taking care of each patient and how to contact them) On call coverage for evenings and overnight, including admissions from the emergency department	“Easier access to consults with internal medicine, where you don't have to wait a week to get a consult and you can work together on patients” “I think there is potential pitfalls with the hospitalist system. If you are transferring a patient out you have to ensure your communication is impeccable with the family physicians. I think there is always room for improvement with that. When you're the family physician discharging your patient to your own practice and you're following them up, it's a lot easier to keep that continuity going” “It would be nice to know who to call for the admitted patients since right now it can be confusing to know which physician is responsible” “You would have a physician onsite for 24/7 and is very reliable, even on holidays and after hours” “…now having someone on 24/7, that hospitalist at night is actually awake and is usually down in the ER working with new patients. They’re awake and on the ball, which is always good. It wasn’t always like that”
Hospitalist staffing and case load	Dynamic schedules where hospitalist staffing fluctuates in real-time with increases and decreases in patient volume Optimal individual hospitalist workload/ census (e.g. one hospitalist to ten patients) Adequate number of hospitalists per site to avoid burnout	“Increase the amount of hospitalists at the site to have hospitalists see the patients earlier in the day” “We are no longer putting out fires. Time was needed to iron out issues. Things are stable now” “The manpower for our program is finally stable. And everyone is very happy with the work environment” “As more and more GPs retire, the growth in the number of unattached patients increases, so you'll need to grow the numbers of hospitalists”

### Strengths and weaknesses of the hospitalist model

We asked interviewees to identify the strengths of the newly implemented hospitalist services (Table 3). The onsite presence of hospitalists was the most commonly cited feature, followed by improvements in teamwork and knowledge of acute medicine by MRPs. Respondents also indicated that the introduction of hospitalists to the care team had resulted in better collegiality between physicians and other providers, closer relationships and better communication, which in turn led to a better work environment.

**Table 3:** Strengths and challenges of hospitalist programs as reported by interviewees (n = 38)

Identified area of strength	Number of instances (all sites)
Improved on-site presence, accessibility and availability of hospitalists	24
Improved inter-professional communication and collaboration	22
Improved knowledge of, and familiarity with, complexity and multimorbidity of hospitalized patients	18
Improved work culture (i.e. improved environment, inter-professional relationships and collegiality, etc.)	15
Increased willingness to address issues that arise among site administration, leadership and hospitalists	6
Identified area of challenge	Number of instances (all sites)
Hospitalist handover leads to longer length of stay; delayed discharges; reduced continuity of care; disjointed communication with patients	22
Limited willingness for hospitalists to admit patients after-hours, leading to congestion. Often only respond to emergency issues	18
Inefficiency of hospitalists following their patients through the hospital even if transferred to different departments	13
Lack of timely administrative data on key performance indicators (e.g. length of stay, readmission rates, etc.) to inform learning, adaptation, and evidence-based decision-making	7
Hospitalists need to improve communication with community family physicians, from admission to discharge	6

On the other hand, interviewees also identified aspects of their newly implemented hospitalist service that they found challenging and offered potential solutions to mitigate them (Table 3). For example, some felt that weekly hospitalist handovers (often on Mondays) lead to longer length of stay, delayed discharges, reduced continuity of care and disjointed communication with patients. There was a perception that some hospitalists often felt the need to re-run tests and develop their own understanding of patient progress and suitability for discharge. They speculated this could be due to lack of trust between hospitalists, concerns about personal liability, and poor handover communication. Respondents identified use of standardized handover forms, longer hospitalist rotations (e.g. two-weeks in length) and rotating start days for service rotations as potential remedies.

Some interviewees also highlighted lack of timely administrative data to inform learning and adaptation. They explained that their sites lacked data on key performance indicators (e.g. length of stay, readmission rates, etc.) that could provide them with concrete information about performance. Additionally, some interviewees found it challenging when hospitalists demonstrated different levels of knowledge and skills, which they attributed to diverse training backgrounds (e.g. family medicine versus internal medicine training). They recommended that when hiring future hospitalists, site administration should ensure that the candidates would be comfortable managing a range of typical acute clinical presentations.

Finally, some respondents identified a lack of clarity between hospitalists and other providers (e.g. internists and general surgeons) about their respective roles and responsibilities. In particular, they described blurred lines around which providers were to take on specific types of patients and concerns about the potential for ‘cherry-picking’ of patients by some physician specialties who should be taking on more complex patients. They felt that site leadership should support hospitalists and other providers to reach clear agreements about their respective roles and responsibilities and scope of practice.

### Lessons from the implementation experience

Interviewees in our study provided a number of lessons for other organizations contemplating inpatient care transitions. They identified clear and repeated communication as the most important factor in managing the change:

“It’s all about clear communication when change is coming up. We had really big issues in our department because there wasn’t any heads up about the changes, especially for front-line workers who are still expected to facilitate discharges.”

Such communication should outline how the new model will work and what the change means for different providers. It should be sent to all stakeholders impacted by the transition including frontline workers. Interviewees expressed preference for a variety of different communication methods, including in-person meetings, memoranda, web information and email. They also underlined the importance of planning for the transition and setting aside 6–12 months to design the model, recruit and hire sufficient numbers of hospitalists, and organize operational aspects of the transition:

“Change is hard, but if we set aside time to plan and prepare, the chaos will be minimized. You need anywhere from 6 to 12 months lead time to set these models up…if you don’t have enough doctors to begin with then it’s very hard to catch up.”

Additionally, proactive monitoring of key metrics that could provide an early indication that existing inpatient care models may collapse (e.g. monitoring the proportion of FPs in the community with hospital privileges) was highlighted:

“We have to keep an eye on the data. As family docs give up their privileges, we have to be aware of that and that we might have to step up and increase our numbers.”

## Discussion

To our knowledge, our paper is the first effort to systematically evaluate perceptions of hospital administrators and other healthcare professionals about the role of the hospitalist model in the delivery of inpatient care within a Canadian healthcare setting. Additionally, our project is an attempt to explore health system leaders’ opinions about characteristics of an ideal hospitalist service and challenges associated with its implementation.

Despite broad differences in healthcare system organization between Canada and the US, hospitalist programs in both countries emerged around the same time and have followed a similar trajectory in growth and evolution [29]. One of the similarities between the Canadian and US hospitalist models is that in both countries, healthcare organizations need to financially support their hospitalists directly in order to attract and retain physicians. As a result, some healthcare administrators perceive their hospitalist programs as a financial loss and this perspective can impact the way they perceive their hospitalist program’s value proposition [24]. Despite this, in the US there is a general agreement that “hospitalists are here to stay” [17, 18] and healthcare leaders need to focus instead on maximizing their partnership with hospitalist physicians in order to enhance care efficiency and quality.

Our findings suggest that many health system leaders in our organization also have a similar assessment about their hospitalist services in spite of the financial implications of implementing such programs. While there are still a significant proportion of interviewees who identified the “traditional” care model as ideal, many acknowledged that it was unsustainable in practical terms. For most interviewees, the hospitalist model provided the ideal structure for inpatient care in their setting, and they identified a number of attributes associated with the new service line that resulted in improvements in the work environment. This is particularly noteworthy, as in British Columbia hospitalists are compensated by their Health Authorities (institutions that operate hospitals and employ hospitalists) through a salary mechanism. As a result, hospitalist programs can constitute a sizable proportion of expenditures on physician compensation for these institutions. In the past, contract negotiations between hospitalists and payers have resulted in province-wide job action or local disputes [30, 31]. Our results suggest that while changes to the compensation model that minimize the financial burden for health authorities (eg. Enhanced FFS or Alternate Payment Programs funded directly by the government) may alleviate some of these challenges, for many system leaders and frontline providers, day to day concerns for patient care and issues related to quality and timeliness of care are higher priorities.

“We need to tell our story about the impact on patient care. This is the most costly, expensive physician service that comes out of the health authority budget.”

Additionally, interviewees identified a number of important attributes for what they considered to be an optimal hospitalist service, and proposed a number of solutions to mitigate the challenges inherent in the model (such as discontinuity of care). These closely mirrored prior research. For example, the Society of Hospital Medicine (Philadelphia, US) has identified a list of specific attributes and characteristics necessary for high functioning hospital medicine programs [32]. Our evaluation confirms that many of these attributes are also relevant and applicable in a publicly funded healthcare environment such as ours. Moreover, these attributes can provide a framework for developing an accountability structure for the hospitalist service and incentives to mitigate the challenges identified by our interviewees.

Our study has a number of limitations. First, our findings represent the views of individuals form a single regional health authority in British Columbia. While we interviewed a wide range of individuals across three acute care facilities where new programs were implemented, our results may not be generalizable to other acute care facilities within the health authority that have had a hospitalist service for a much longer time period, or other organizations in other parts of Canada. Indeed, there is significant regional variation across Canada in the penetration of the hospitalist model, as well as various characteristics of such programs. A national study would be needed to confirm the applicability of our findings more broadly. Second, while administrators represented the largest group of those interviewed, some of the participants were front line providers who may not have had a similar “systems” view about their newly implemented hospitalist services and the financial implications of transitioning to a new service model. As such they may have prioritized more day-to-day aspects of inpatient care processes in their evaluation. Finally, most of our respondents held low to mid-level leadership positions, and their views may not represent those of system leaders with different levels of responsibilities within the health system leadership hierarchy.

## Conclusions

The majority of hospital administrators and front line professionals interviewed at three facilities with newly implemented hospitalist services within our regional health system identified the hospitalist model as the ideal inpatient care delivery mechanism. They also identified a number of key features that should be incorporated into the design and implementation of hospital medicine programs. For example, ensuring that clinical rotations maximise continuity of care and streamlining the handover process was deemed critical by many respondents. While the majority of respondents found the implementation of the program had resulted in a number of improvements, they acknowledged that the hospitalist model was also associated with a number of challenges, and proposed a number of concrete solutions for overcoming these shortcomings.

## Authors’ information

Elayne McIvor completed her Masters of Public Health (Global Health Concentration) at Simon Fraser University. Her capstone research focused on exploring the outcomes of participating in support programs for women living with HIV in British Columbia from a qualitative perspective. She has conducted a wide range of research and evaluation projects within the non-profit, governmental and private sectors that address a range of public health issues, such as HIV/AIDS, mental health and addictions, Aboriginal health, chronic diseases, and women's health.

Vandad Yousefi is a hospitalist in Vancouver, British Columbia. Upon receiving his medical degree from the University of British Columbia in 2004, he moved to Calgary to complete his family medicine residency. He has published a number of peer reviewed articles examining the impact of Hospitalist programs across Canada, and has been invited to speak at national and international conferences on related topics.

## Data Availability

Data for this study can be requested by contacting the corresponding author, and will be provided contingent on approval from the Fraser Health Authority.

## Abbreviations

LOS: Length of Stay
US: United States
FP: Family Physician
MRP: Most Responsible Provider
FFS: Fee for Service
FH: Fraser Health
NP: Nurse Pracatitioner
GP: General Practitioner
